# Behavioural Signs of Pain in Cats: An Expert Consensus

**DOI:** 10.1371/journal.pone.0150040

**Published:** 2016-02-24

**Authors:** Isabella Merola, Daniel S. Mills

**Affiliations:** School of Life Sciences, University of Lincoln, Joseph Banks Laboratories Green Lane, Lincoln LN6 7DL, United Kingdom; GI Lab, UNITED STATES

## Abstract

**Objectives:**

To identify where a consensus can be reached between veterinary experts in feline medicine on the core signs sufficient for pain (sufficient to indicate pain when they occur, but not necessarily present in all painful conditions) and necessary for pain (necessary in the presence of pain, but not always indicative of pain).

**Methods:**

A modified Delphi technique was used, consisting of four rounds of questions and evaluation using nineteen participants during the period December 2014 and May 2015. Agreement was considered to be established when 80% of the experts concurred with the same opinion.

**Results:**

Twenty-five signs were considered sufficient to indicate pain, but no single sign was considered necessary for it.

**Discussion:**

Further studies are needed to evaluate the validity of these 25 behavioural signs if a specific pain assessment tool is to be developed that is capable of assessing pain in cats based on observational methods alone. The signs reported here may nonetheless help both vets and owners form an initial evaluation of the pain status of cats in their care.

## Introduction

Pain is a multidimensional experience involving far more than mere sensation [1]. It has primarily two domains: the sensory aspect (intensity, location and duration) and the affective-motivational (emotional and unpleasantness). Its assessment in cats has been investigated using a variety of methods, such as behavioural and physiological scales, provoked tests or natural observations,[2–4] in a range of clinical contexts such as following different surgical procedures or in relation to orthopaedic disease, etc. [5] Interest in this subject is not only important from an academic perspective, but also and perhaps primarily for its practical value to veterinarians and owners who need to recognise the signs of pain in this species in order to reduce suffering [6].

To assess pain, a tool based on natural observation seems preferable to minimise suffering, although interacting with the painful animal can be important when detecting pain (e.g. palpating the affected area) [7]. Observation of behaviour is a non-invasive and effective way by which pain can be easily investigated by different people [8], in different contexts (e.g. at home, or in the clinic) with none of the risks (for either the cat or observer) implicit from closer interaction. There is a need for a well-validated cat pain behavioural assessment tool that could be potentially used by both veterinary experts and owners/caregivers.

Even though individuals vary in the features they attend to when assessing pain such as which behavioural features they consider most important [9], or the ability to use specific methods, such as diagnostic tests, both owners and veterinarians are clearly able to recognise many relevant changes in cats related to pain^4^. However, owners may not always recognise the clinical relevance of all the observations that they make [9]. For example, they may view them as an inevitable part of the natural ageing of the animal [10] and not report them to the vet as a concern or at least not until they become quite severe. The ability to recognise behavioural indicators of pain may depend on familiarity with and knowledge of the species by the assessor [11], and may be subjective and biased if objective criteria are not used to define and evaluate these observations [12]. More objective criteria that relate to specific signs of pain might improve the ability of both owners and vets to recognise it. Furthermore, although single behaviours might be suitable for simple presence/absence observations, the use of a combination of behaviours can result in greater accuracy [11].

A recent systematic review [13] of instruments for assessing feline pain concluded that, at present, only one tool (the UNESP-Botucatu) has a reasonable degree of validity and reliability (see [13] for an explanation of these terms), but this was only established within the context of post-ovariohysterectomy. In addition to natural observation, this scale involves behavioural observation after a provoked reaction and the evaluation of physiological parameters [5]. Other feline pain assessment tools are undergoing a validation process [14,15], but until a tool is fully validated, the outputs arising from the use of this tool may only reflect preconceived ideas of what a painful cat may look like.

Even though no tool based on natural observation has been validated, three studies have focused on creating a pool of naturally observable behaviours, which may be a reliable expression of pain in cats [6,16,17]. Both Cloutier et al. [16] and Waran et al. [6] looked at the frequencies of certain behaviours shown by cats when in pain (in a randomised controlled trial with negative control group post-ovariohysterectomy or tenectomy/onychectomy). These authors highlighted that some behaviours, such as crouching or shaking of the forepaws, are expressed more often in “painful” cats than in control subjects. Furthermore, Holden et al. [17] compared pictures of the facial expressions of cats in pain (for a wide range of painful conditions) with a different group of cats not in pain. Analysing the distances between landmarks on the faces, the authors found that cats in pain have a greater distance between the ears and a shorter nose/muzzle distance compared to the control group. However, although picture angles were similar, they were not exactly the same and different subjects were observed for the two conditions (in pain and not in pain). Either or both of these factors might result in morphometric error accounting for the differences observed between the two groups. Other behavioural signs have also been reported in the literature with some evidence to support them (see Merola and Mills [13] for a review and also [18,19]) such as lameness, abnormal gait, difficulty to jump, modification in human-cat interaction, increased or decreased grooming and changes in overall activity, appetite/food intake as well as alteration in the general mood of the subject. As noted previously, these signs need to be clearly and unambiguously defined and may need to be used in combination with both other behaviours and the wider context to be accurate indicators of pain. However, there could be specific behaviours, which are reliable indicators of pain on their own whenever they occur, i.e. they are sufficient for making the inference of pain. However, that is not to say that such signs must occur when an animal is in pain, i.e. they are not necessary signs of it. In the absence of good scientific evidence about the value of specific signs as markers of pain [20,21], there is a danger that their value is based solely on the subjective opinion of the observer [11]. Nonetheless there is a need to establish an initial list of candidate behavioural signs related to pain to be prioritised for future validation. In the absence of definitive objective evidence for any sign, expert opinion and judgment provides a reasonable starting point [22] for identifying the signs which should be prioritised, especially if the opinion of several experts come to the same conclusion.

Therefore, our aim was to collect and classify expert opinion on the possible behavioural signs in cats that denoted pain in general, according to whether they were sufficient (i.e. their presence indicates that the cat is in pain) and/or necessary (i.e. their presence is essential to indicate pain, but not necessarily pathognomonic for pain) signs of either low or high level pain in this species. The severity or intensity of pain in this context is difficult to define or quantify and may be influenced by one’s own experience, but that does not negate the value of identifying where a consensus can be built among veterinary experts. By creating a core set of signs, we lay the foundation for future studies aimed at creating pain scales for cats based on natural observation that minimise subjective bias in the interpretation of each sign. To do this, we listed the behavioural signs of pain highlighted previously in our systematic review of the literature [13] and contacted veterinary experts in different disciplines of feline medicine to ask their opinion about the reliability of these behavioural signs as indicators of pain.

A modified Delphi method (following the guidelines of Hsu and Sandford [23]) was used in order to try to build consensus. This technique uses a series of interactions to adjust the initial opinion of each participant, based on the feedback received from the whole group in order to establish maximal agreement on a topic [24].

## Materials and Methods

The process to develop a consensus around the behavioural expression of pain in cats started in December 2014 and ended in May 2015. The systematic review of behavioural assessment of pain in cats, previously conducted by the authors, was used to provide initial signs (67 behaviours) for consideration [13].

### Ethics statement

This project was approved by the delegated, Life Sciences, University of Lincoln ethics committee.

### Participants

A panel of international veterinary experts in feline medicine was invited to contribute to the study. Members were initially selected on the basis of their authorship of relevant papers included in the systematic literature review (based on articles from between 2000 to February 2014) or the recommendation of these authors, with the intention of involving experts from a wide range of disciplines and from different countries. Areas of interest were: internal feline medicine, oncology, anaesthesiology, behaviour, orthopaedics, cardiology, dermatology, dentistry, ophthalmology, gastroenterology and neurology. Criteria for selection of an expert were further refined on the basis of their clinical experience in feline medicine.

Experts were contacted by email, and the aim of the study with a general introduction to the project included. Experts could recommend other experts in the field (snowball sampling method), and if names were given, these individuals were contacted in the same manner. A consent form and a general information questionnaire were sent to the experts to confirm their interest in participating. The general information questionnaire included items on: gender, age, type of clinical practice (private or academic), country of work, specialism, years of clinical practice and the number of cases seen in a month where the cat was being evaluated for a painful condition. Once the participants had returned the form, the first exercise was sent to them by email. Because many studies suggest value in having a final sample in the last Round of about 11–25 participants (see [24] for a systematic review on Delphi technique) an initial population of 20 experts was targeted.

### Consultation procedure

The procedure aimed to reach a consensus through a structured process of consultation rounds. This can be obtained in two ways: it can continue until consensus is reached or it can be structured around a previously determined number of rounds. In this study, we decided a threshold of four rounds for the process in the absence of consensus: as highlighted by Hsu and Sandford [23] three interactions are in the majority of the cases sufficient to collect the needed information and to reach a consensus in most cases.

Traditionally, the first Round of the Delphi method starts with an open-ended questionnaire [23]; however, because we already had a list of signs related to pain expression in cats from the systematic review [13], we proposed a more structured survey. In order to reduce the bias related to a predefined list of behaviour, we added to this list some signs which provided a contrast to some of those evidenced in the systematic review [13] (e.g. if “sleeping more” was identified as related to pain by the review [13], we added “sleeping less” as well, to have a wider range of signs to evaluate). Thus the review provided the type of sign but not necessarily its quantification. Furthermore, in order to avoid losing important information at this time, we gave participants the opportunity to write comments about the signs provided and to add new behaviours that they thought were relevant to the identification of pain in cats. Participants were required to answer the questions based on their knowledge and professional experience.

In the first part of the survey we asked the experts to list up to 10 conditions that they believed were reliably and inherently painful to cats: this enabled us to gain a perspective on the conditions they were referring to when replying to the survey.

Then we reported 67 behavioural signs related to pain and we asked participants to evaluate these according to 4 different properties for each sign:

1. if the sign was present in acute and/or chronic conditions and/or non-painful conditions,
2. their opinion on the reliability of the sign as an indicator of pain (certainly/often reliable or little reliability/unreliable), i.e. its sufficiency as a sign of pain
3. how likely it was that the sign was present when there is a low level of pain (frequently/sometimes or rarely/never)
4. how likely it was that the sign was present when there is a high level of pain (frequently/sometimes or rarely/never)

Finally we asked participants to add any behavioural expressions of pain overall that they could think of that were not included in the list, but relevant to them based on their practical clinical experience, not only referring to cases examined during the Delphi process.

The subsequent 3 Rounds were built on this structure following the participant’s response. In Round two we added the signs reported in Round 1 by the experts as expressions of pain in cats. Furthermore, in Rounds 2, 3 and 4 for each sign we reported the percentage of individuals that agreed with the modal response and the previous answer of each participant to themselves but no-one else, so that they could see how the majority replied and to re-evaluate their previous answer.

The experts had 2 weeks to reply for each Round: if during these two weeks a participant asked for a longer period, this was subject to negotiation with the authors, taking account of the need for all participants to be working at the same stage of the process. If no specific request was made the participant was excluded, even if their reply was subsequently received late.

After the 4th Round, results were sent to the participants in order to get final comments, to assist with the interpretation of the signs (especially where there was disagreement), and to identify if any sign was considered necessary (essential even if not pathognomonic) for the occurrence of pain.

### Data analysis

All the answers were transcribed into an Excel file and percentage agreement for each question was calculated (as recommended by Diamond et al. [24]). Agreement was considered to be established if 80% of the experts reported the same answer for all 4 aspects of each sign. If agreement was not achieved the sign was presented again to the participants in the next Round, with the opportunity to comment on those aspects where agreement had not been obtained. All comments were analysed and discussed between the authors, with modification made as appropriate. Agreements and disagreements were reported in percentages.

## Results

### Participants

The recruitment process was as follows: a total of 50 participants were invited to take part; of these 19 agreed to participate and completed the consent form and the general information questionnaire. Of these, 10 were female and 9 were male, ages ranged from around 30 to 60 years old. Six experts were working as private clinicians, while thirteen were academic staff. Their specialism varied from internal medicine to anaesthesiology, oncology, dentistry, behaviour, dermatology, ophthalmology and neurology (no cardiologist agreed to take part in the study). Three of them were working in clinical practice for less than 5 years and all the others had been practicing for more than 10 years. All the participants were working and treating cats with painful conditions with an average of 10 cats in pain seen by each individual per month.

### Rounds

Nineteen experts responded within the required timeframe to Round 1, seventeen to Round 2, sixteen to Round 3, fifteen to Round 4. After Round 4, when results were sent to the participants in order to get their final comments and identify if any sign was considered necessary for the occurrence of pain, ten out of the nineteen participants replied.

The 10 diseases each of the 19 experts chose to list for referring to when considering signs of pain can be summarised thus: 15 referred to orthopaedic conditions (such as osteoarthritis, degenerative joint disease and fractures), 13 to cancers (in particular to osteosarcoma), 13 to problems related to the urinary tract (such as cystitis and obstructions of the ureters and urethra), 10 to pancreatitis, 9 to ophthalmic conditions (e.g. uveitis), 8 to dental and oral conditions (e.g. dental fracture, stomatitis, gingivitis), 8 to general trauma, and 8 to surgical pain. A minority of participants (less than 5 for each disease) referred also to peritonitis, diabetes, bowel disease, foreign body ingestion, vertebral disc disease, thromboembolism, neuropathic pain, skin damage and dermatological conditions such as burns, wounds and ear infections, visceral inflammation, oro-facial pain syndrome, bites and cat fights.

**Fig 1:**
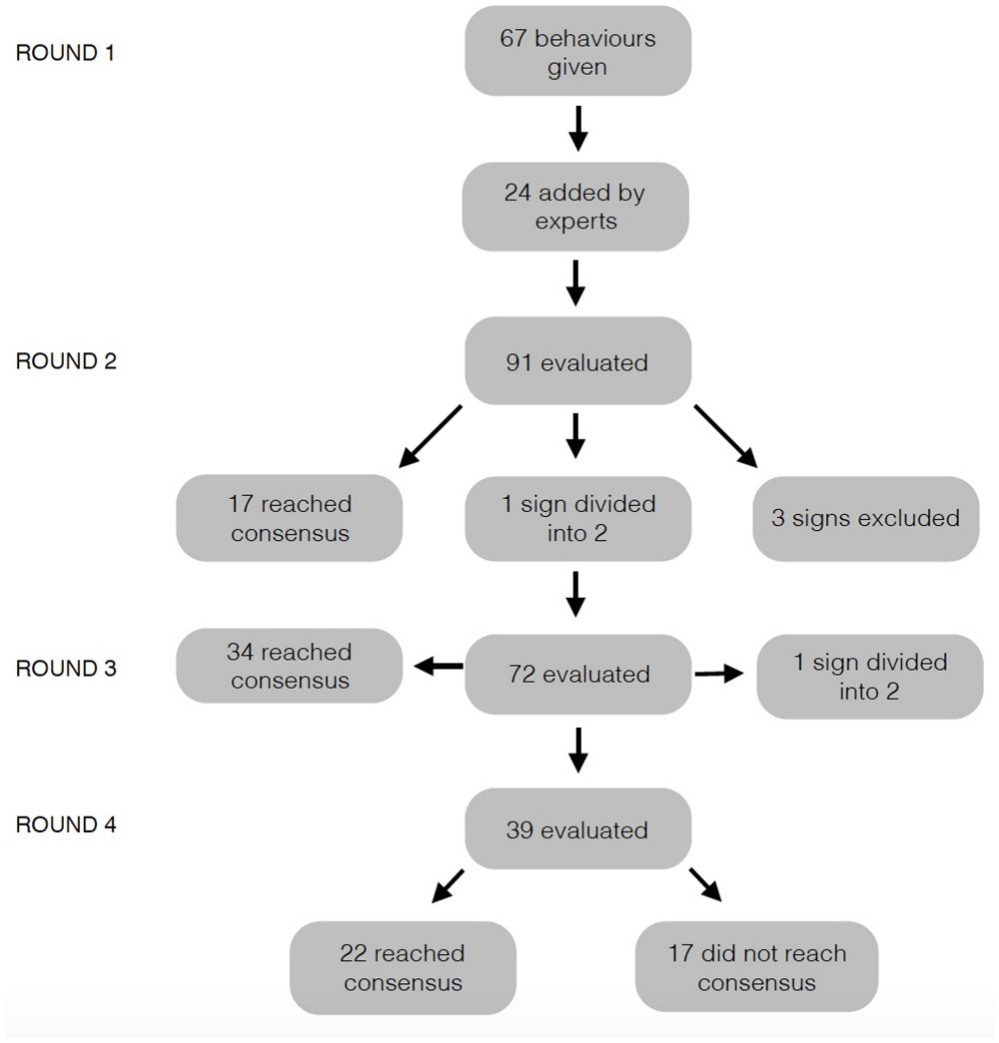
Diagram of the number of signs per Round and number of agreed upon signs per Round.

In Round 1 (see Fig 1) the experts scored 67 behaviours (see Appendix 1), but no agreement was reached for any of them. In this Round the participants highlighted the need to clarify some of the signs (sitting, rolling skin syndrome, mood, temperament), and this was addressed in the next Round (e.g. sitting longer than usual, the definition of mood state as an enduring episodic change in underlying affective predisposition arising as a result of a series of emotional events of congruent emotional valence, for example, a tendency to be irritable from time to time as a result of pain) [25].

In Round 2 twenty-four new signs (see Appendix 2) were added to extend the list to 91 signs. After this Round the experts had reached a consensus on 17 of them. The participants also highlighted difficulty in scoring three of the 24 new signs and so these behaviours were excluded from the next Round (“tension around the eyes and muzzle”, “low grade tail flicking” and “lips drawn down back”). Furthermore experts asked to divide the sign “change in rate (e.g. eating more quickly or more slowly)” into “change in rate: eating more quickly” and “change in rate: eating more slowly”.

In Round 3 the experts scored the remaining 72 items and an agreement was reached for 34 of them. In this Round a request to clarify the focus of the sign “scratching more” was made; and so this item was divided into “scratching objects more” and “scratching self more”.

In Round 4 the remaining 39 signs were scored: for 22 of these the experts reached agreement, leaving 17 without consensus.

Of the total 73 signs where an agreement was reached, 23 items were considered as certainly or often sufficient to establish the occurrence of pain (see Table 1), while 36 items were considered not sufficient for pain, but possibly present in low or high level of pain (see Table 2). Finally 14 signs were considered not sufficient to infer pain and never or rarely present in both low or high level pain (“playing more”, “lying on its back”, “whiskers raised”, “more rubbing toward people”, “more rubbing toward objects”, “shaking feet”, “hiding down in the sink”, “vomiting”, “overall activity increasing”, “pupil constriction”, “scratch object more often”, “seeking contact with a person more often than usual”, “body relaxed and tail up”).

**Table 1:** Behaviours considered by participants as sufficient (reliable) for pain and their presence in high and/or low level pain.

Behaviour sufficient for pain	Presence in low level pain	Presence in high level pain	Participant comments
Lameness	Frequent	Frequent	
Difficulty to jump	Frequent	Frequent	
Abnormal gait	Frequent	Frequent	Can be provoked by other conditions: e.g. cerebellar hypoplasia
Reluctant to move	Frequent	Frequent	
Reaction to palpation	Frequent	Frequent	
Withdraw/hiding	Frequent	Frequent	
Absence of grooming	Frequent	Frequent	
Playing less	Frequent	Frequent	
Appetite decrease	Frequent	Frequent	
Overall activity decrease	Frequent	Frequent	
Less rubbing toward people	Frequent	Frequent	
General mood ^1^	Frequent	Frequent	
Temperament ^2^	Frequent	Frequent	
Hunched up posture	Frequent	Frequent	
Shifting of weight	Frequent	Frequent	It is relatively subjective
Licking a particular body region	Frequent	Frequent	
Lower head posture	Frequent	Frequent	
Blepharospasm*	Frequent	Frequent	Caused by any chronic eye disease
Change in form of feeding behaviour	Rare	Frequent	Require extensive knowledge of prior feeding behaviour. Not reliable to pain
Avoiding bright areas	Rare	Frequent	Any disease of the eyes can cause it
Growling	Rare	Frequent	More useful if it is a new behaviour, related to mood
Groaning	Rare	Frequent	Not reliable sign of pain
Eyes closed	Rare	Frequent	Other possible causes for it (not specified)

* this behaviour was considered reliable for an acute condition.

^1^ Mood states: i.e., enduring episodic changes in underlying affective predisposition arising as a result of a series of emotional events of congruent emotional valence, for example a tendency to be irritable from time to time as a result of pain^25^

^2^ Temperament, i.e., a general disposition or trait that is consistent across time and contexts. This indicates that the pain is persistent, or relief is only temporary, and that a state of pain has become an integral part of the animal’s constitution and its behavioural predispositions shifted accordingly to adapt to the impact of this. For example, a cat in chronic pain might be described as jumpy or irritable the whole time^25^.

**Table 2:** Behaviours considered by participants as not sufficient (unreliable) to infer pain, but shown by cats in high and/or low level pain.

Behaviour not sufficient for pain	Presence in low level pain	Presence in high level pain	Participant comments
Sitting more often	Rare	Rare	Difficult to evaluate
Rolled up	Rare	Frequent	Associated with fear and stress
Standing longer than usual	Rare	Rare	
Lying on its side	Rare	Frequent	
Crouching	Rare	Frequent	
Body tense	Frequent	Frequent	
Hissing	Frequent	Frequent	More related to cat temperament
Meowing	Rare	Frequent	
Crying	Rare	Frequent	
Half blink	Rare	Frequent	
Pupil dilation*	Rare	Frequent	
Ear rotated	Rare	Frequent	
Ear downward	Rare	Frequent	
Ear flattener	Frequent	Frequent	Sign of fear
Panting*	Rare	Frequent	
Seeking contact with a person	Frequent	Frequent	Depends on personality
Less rubbing on objects	Rare	Frequent	Sign of distress
Over grooming	Frequent	Frequent	
Teeth grinding	Rare	Rare	Could be related to altered chewing pattern. Rare sign in cats
Trembling or shivering	Rare	Frequent	Could be associated with fear and stress. Rare sign in cats
House soiling	Rare	Frequent	Maybe indicative of change in mobility
Spitting	Rare	Frequent	
Purring	Rare	Rare	
Tongue showing**	Rare	Frequent	
Mouth semi open	Rare	Frequent	More a respiratory cardiac sign
Hiding down in the litter box	Rare	Frequent	General sign for systemic illness in cats
Escaping when trying to catch	Rare	Frequent	Important if is a change in the normal behaviour
Trying to scratch someone	Rare	Frequent	
Trying to bite someone	Rare	Frequent	
Reduction urination	Rare	Frequent	
Appetite increase	Rare	Rare	Is not a sign of pain
Scratching less	Rare	Frequent	
Sleeping more	Frequent	Frequent	
Sleeping less	Rare	Frequent	
Lying ventrally	Frequent	Frequent	Is not a sign of pain

* the consensus was that these behaviours were related more to acute conditions, but that they were not reliable indicators of pain.

** this behaviour was not related to either acute or chronic pain, but considered as present in high level pain, all the others were related to both (acute and chronic) situations.

Although 80% agreement was reached for these signs, some participants reported further comments on some signs: these are included in Tables 1 and 2.

The 17 behaviours where the experts were in disagreement on one or more of the 4 properties of the sign (i.e. agreement on all aspects of the sign was not reached), had the following characteristics:

- 2 (“straining to urinate” and “tail flitching”) were considered sufficient to infer pain, but a consensus was not reached on their frequency during either low or high level pain;
- 13 (“licking objects or people”, “whisker downwards”, “whisker backwards”, “mouth open”, “hyper salivation”, “rolling skin syndrome”, “furrowed brow”, “vocalizing when yawning”, “third eyelid shown”, “change in rate eating more quickly” and “change in rate eating more slowly”) were not considered sufficient to infer pain and rarely present in low level pain, but no agreement was reached with regard to their frequency in high level pain or their presence in acute, chronic, both or no pain conditions;
- For the final 4 signs (“Scratching self more often”, “Increase respiratory rate”, “Vocalizing when yawing”, “Change in dietary preference”), the experts were not in agreement about the sufficiency of these behaviour in relation to pain. The first three signs were considered rare in a low level of pain, but no agreement was reached for high level pain, while “change in dietary preferences” was considered to be as frequently present in low as high level pain.

After the 4^th^ Round, once the consensus on the sufficient signs had already been reached: 10 of 15 participants replied to our final request and none reported any behaviour to be a necessary sign of pain.

## Discussion

A Delphi method involving veterinary experts in feline medicine was used in this study, in order to create a core collection of signs identified as necessary and/or sufficient for pain assessment in cats. Different specialisms were involved in order to create a wide overview of painful phenomena. Although the use of a variety of veterinary specialists (working with cats daily) may have hindered consensus on some signs, the variety increased the likelihood that where there was a consensus that these signs were universal signs of general pain in cats, that can be used by both clinicians and other observers such as owners if adequately defined. It is also worth emphasising that participants had an opportunity to add to the initial list taken from the literature based on their experience with cats in pain. These additional signs were then appraised by the other participants, creating a wider list of behaviours that included signs related to a wide range of conditions.

This consensus building exercise took over 5 months to complete, as we needed to give participants sufficient time to evaluate each sign, recognising their co-commitment to their other professional responsibilities. This evaluation involved them reflecting on the signs in light of their previous experience and knowledge as well as the other participants’ opinion.

A total of 91 signs were collated and assessed in this exercise. Despite the high number of signs and the fact that for each of them 4 different aspects had to be evaluated by participants, a high level of consensus was built since only seventeen behaviours were left with some disagreement in the final Round, with no sign failing to gain consensus on at least one aspect. It might be that the decision to limit consultation to a maximum of 4 Rounds played a role in the failure to reach agreement on some of these seventeen items, since this list included 10 of the 24 signs added by the experts during the first Round and these signs were subject to only 3 Rounds of consultation. However, we needed to define and limit the workload of the experts from the outset in order to maximise the prospects of their engagement with the project.

Only 3 signs were reliably linked to acute pain (panting, pupil dilation and blepharospasm), and no signs were specifically related to chronic conditions (although the majority of participants related blepharospam to acute conditions, one participant suggested it could occur in chronic painful conditions as well, as mentioned in Table 1). On the one hand these results might evidence the possible presence of a common suite of behavioural signs to both acute and chronic conditions, but on the other hand this might highlight the difficulty involved in reliably distinguishing between these two conditions in a clinically meaningful way, as already noted in our systematic review [13]. In fact although three “acute” signs were highlighted, a non-specific description of acute was given (a more recent (acute) or more intense (acute) situation), based on common usage, leaving open the question of the clinical usefulness and reliability of this distinction.

None of the 91 signs was considered necessary to denote pain (i.e. if this sign is absent we cannot consider the subject to be in pain), supporting the idea that evaluation of a suite of behaviours will produce greater accuracy compared to the use of single signs, especially across different contexts [12].

For pain assessment to have validity, any tool based only on behavioural expression should consider both the sensorial and affective domains of pain [13]. The 23 signs considered sufficient to infer pain (i.e. if this sign is present the subject is in pain, but its absence does not exclude the presence of pain), plus the two behaviours (“straining to urinate” and “tail flitching”) considered reliable for pain, (but with disagreement on the intensity of pain being experienced) cover both the sensorial aspects (e.g. signs such as lameness, difficulty in jumping, etc.) and the emotional aspects (e.g. changes in general mood and temperament) of pain. These therefore give a useful starting point for future validation with a view to providing a more comprehensive evaluation of subjects.

The majority of behaviours indicated by the experts as reliable indicators of pain are in accordance with those described in the WSAVA guidelines for the assessment of pain [18], but in our results (unlike the guidelines) they were not specifically linked to acute or chronic conditions. In addition, some signs described in the guidelines were not considered in our study to be reliable signs of pain (e.g. furrowed brow, sitting or lying in abnormal positions). Nevertheless, although agreement was reached, some experts (probably included in the 20% not in agreement) had further comments on these signs, such as the need to know the general behaviour, mood and temperament of the individual subject being evaluated (e.g. increased growling is reliable for pain, but strongly related to the cat’s mood). Other comments highlight that some signs may not be reliable for pain since they may be part of the expression of a specific other problem e.g. neurological condition which is not considered painful. These comments provide useful refinement to the consideration of these signs. The importance of a thorough understanding of mood and temperament in the detection of pain was also highlighted by the responses, because both were included as reliable signs of pain, but they are also linked to the expression of other signs (e.g. growling). It seems that there is potentially great value to be gained by including reference to these aspects in any evaluation. However it is important that any proposed instrument including these aspects is informed by a solid current understanding of the science related to emotional states in non-human animals. Personality, temperament and mood seem to influence the way cats show pain and specific tools to assess these specific traits would be helpful. Additional investigation is also needed to determine the ability of individuals to read the expressions of cats. Although there seems to be generally good agreement, there is evidence to suggest that untrained observers tend to greatly anthropomorphise the emotions of cats, ascribing them overly complex human emotions and motivations [26].

Finally, some signs evidenced by the literature as related to pain, e.g. by Cloutier et al. [16] (shaking feet and crouching behaviour identified in cats after tenectomy/onychectomy), were not considered by participants to be reliable signs for pain in general. They were in fact, considered to be an expression of other clinical conditions and not specific to pain, although crouching was reported to be associated with a high level of pain, when it was related to this problem. This last point emphasizes the importance of considering first the context and then intensity when assessing pain; because, although in certain conditions (e.g. after tenectomy/onychectomy) these behaviours may be expressed more frequently by subjects in pain; it seems, based on our results, that these signs are not generalizable to unspecified clinical contexts. This, once again, cautions against the use of a specific sign to denote pain in general for cats.

Given that our aim was to highlight the sufficient signs of pain that can be observed non-intrusively in cats with the ultimate goal of creating and validating a behavioural assessment tool for this species that could be useful in a range of painful conditions, the signs found in our results seem to be a good starting point, and may be of wider value not only to clinicians trying to assess pain in cats but also to owners trying to determine if their cat is in need of veterinary attention. However, further studies are necessary to evidence their validity when assessing pain in cats provoked by different conditions, and also to evaluate their reliability when used by untrained observers (e.g. owners).

## Conclusion

Twenty-five behavioural signs were considered by experts to be reliable and sensitive for the assessment of pain in cats, across a range of different clinical conditions. Some of these signs have been highlighted in previous scientific literature, but some arose from the experience and knowledge of experts. These results improve our knowledge of this topic, but further studies are necessary in order to evaluate their validity and clinical feasibility (especially in relation to different intensities of pain) to help vets and caregivers of cats recognize pain in this species effectively and as early as possible to maximise cat welfare.

## Appendix 1

List of 67 behaviours proposed at the Round 1

1. Abnormal Gait,
2. Absence/reduction of grooming,
3. Appetite/feed intake decrease,
4. Appetite/feed intake increase,
5. Body relaxed,
6. Body tense,
7. Crouching,
8. Crying,
9. Difficulty to jump (up and down),
10. Ear downward,
11. Ear flattener,
12. Ear rotated,
13. Escaping when owner tries to catch it,
14. Eyes closed,
15. General mood state,
16. Groaning,
17. Growling,
18. Half blinks (eyes half closed),
19. Hiding/lying down in the bath/ sink,
20. Hiding/lying down in the litter box,
21. Hissing,
22. House soiling,
23. Lameness,
24. Less rubbing on objects,
25. Less rubbing toward people,
26. Licking objects or people,
27. Lying on its back,
28. Lying on its side,
29. Lying ventrally (on the stomach),
30. Meowing,
31. More rubbing on objects,
32. More rubbing toward people,
33. Mouth open,
34. Mouth semi open,
35. Over Grooming,
36. Overall activity/movement decrease,
37. Overall activity/movement increase,
38. Panting,
39. Playing/hunting less,
40. Playing/hunting more,
41. Pupil constriction,
42. Pupil dilation,
43. Purring,
44. Reaction to palpation,
45. Reduction urination and defecation,
46. Rolled up,
47. Scratching less,
48. Scratching more,
49. Seeking contact with a person less often than usual,
50. Seeking contact with a person more often than usual,
51. Shaking feet,
52. Sitting more often,
53. Sleeping less,
54. Sleeping more,
55. Spitting,
56. Standing longer than usual,
57. Tail down,
58. Tail up,
59. Temperament
60. Third eyelid shown,
61. Tongue showing,
62. Trying to bite someone,
63. Trying to scratch someone,
64. Whiskers backwards,
65. Whiskers downwards,
66. Whiskers raised,
67. Withdrawn/Hiding

## Appendix 2

List of 24 behaviours added at the Round 2

1. Avoiding bright areas/photophobia,
2. Blepharospams,
3. Change in dietary preference (e.g preferring soft or other specific food),
4. Change in form of feeding behaviour (e.g. gulping food, eating on one side),
5. Change in rate of eating—more quickly or more slowly
6. Furrowed brow,
7. Hunched up posture (when standing or moving),
8. Hypersalivation,
9. Increase respiratory rate,
10. Licking or biting a particular body region or damaged area,
11. Lips drawn down, back,
12. Low grade tail twitching,
13. Lowered head posture,
14. Reluctance to move,
15. Rolling skin syndrome,
16. Shifting of weight,
17. Straining to urinate,
18. Tail Flicking,
19. Teeth grinding,
20. Tension around the eyes and muzzle,
21. Trembling or shivering,
22. Vocalizing when yawning,
23. Vocalizing while eating,
24. Vomiting,

## Supporting Information

S1 Table Results of the four rounds are reported as percentage of agreement between experts. Highlighted in pink the behaviours that reached an agreement for the four property of the sign, in red the behaviours that experts excluded from the process. In order to guarantee anonymity, experts have been indicated as participants 1, 2 etc.
(XLSX)
